# The Feasibility Study of Non-Invasive Fetal Trisomy 18 and 21 Detection with Semiconductor Sequencing Platform

**DOI:** 10.1371/journal.pone.0110240

**Published:** 2014-10-20

**Authors:** Young Joo Jeon, Yulin Zhou, Yihan Li, Qiwei Guo, Jinchun Chen, Shengmao Quan, Ahong Zhang, Hailing Zheng, Xingqiang Zhu, Jin Lin, Huan Xu, Ayang Wu, Sin-Gi Park, Byung Chul Kim, Hee Jae Joo, Hongliang Chen, Jong Bhak

**Affiliations:** 1 TheragenEtex Bio Institute, Suwon, Republic of Korea; 2 Genome Care, Seoul, South Korea; 3 Molecular Diagnostics Laboratory, Department of Medical Genetics, Prenatal Diagnosis Center, Xiamen Maternal and Child Health Hospital, Xiamen, Fujian, China; 4 Xiamen Hospital of Traditional Chinese Medicine, Fujian University of Traditional Chinese Medicine, Xiamen, Fujian, China; 5 Xiamen Vangenes BioTech, Xiamen, Fujian, China; 6 Zhangzhou Affiliated Hospital of Fujian Medical University, Zhangzhou, Fujian, China; 7 Clinomics, Ulsan, Republic of Korea; 8 Personal Genomics Institute, Genome Research Foundation, Suwon, Republic of Korea; 9 BioMedical Engineering, UNIST, Ulsan, Republic of Korea; University Hospital Basel, Switzerland

## Abstract

**Objective:**

Recent non-invasive prenatal testing (NIPT) technologies are based on next-generation sequencing (NGS). NGS allows rapid and effective clinical diagnoses to be determined with two common sequencing systems: Illumina and Ion Torrent platforms. The majority of NIPT technology is associated with Illumina platform. We investigated whether fetal trisomy 18 and 21 were sensitively and specifically detectable by semiconductor sequencer: Ion Proton.

**Methods:**

From March 2012 to October 2013, we enrolled 155 pregnant women with fetuses who were diagnosed as high risk of fetal defects at Xiamen Maternal & Child Health Care Hospital (Xiamen, Fujian, China). Adapter-ligated DNA libraries were analyzed by the Ion Proton™ System (Life Technologies, Grand Island, NY, USA) with an average 0.3× sequencing coverage per nucleotide. Average total raw reads per sample was 6.5 million and mean rate of uniquely mapped reads was 59.0%. The results of this study were derived from BWA mapping. Z-score was used for fetal trisomy 18 and 21 detection.

**Results:**

Interactive dot diagrams showed the minimal z-score values to discriminate negative versus positive cases of fetal trisomy 18 and 21. For fetal trisomy 18, the minimal z-score value of 2.459 showed 100% positive predictive and negative predictive values. The minimal z-score of 2.566 was used to classify negative versus positive cases of fetal trisomy 21.

**Conclusion:**

These results provide the evidence that fetal trisomy 18 and 21 detection can be performed with semiconductor sequencer. Our data also suggest that a prospective study should be performed with a larger cohort of clinically diverse obstetrics patients.

## Introduction

Prenatal screening and diagnostics for fetal chromosomal aneuploidy are well established worldwide for pregnant women [1], [2]. Predictive testing during the first trimester includes a combination of fetal ultrasound and maternal serum biomarkers [3]. Women with fetuses at high risk of defects are also provided opportunities for invasive diagnostic testing, such as chorionic villus sampling (CVS) at 12 weeks of gestation and amniocentesis at 15 weeks of gestation [4], [5]. Although these invasive tests are highly accurate, they are associated with iatrogenic pregnancy loss [6].

The prevalence rate of the common aneuploidy, trisomy 21, is around 1 in 500 in the group of women that request screening. In contrast, 1 in 200 women who receive first-trimester screening (FTS) are categorized as increased risk. The main reason that many women avoid testing or screening is because they are aware of the risks associated with invasive testing [7]. Further, FTS has two serious limitations; FTS has a false-negative rate of 10–25% and a restricted time-window of 11–13 gestational weeks [8]–[12].

After Lo et al. discovered fetal cell-free DNA (cffDNA) floating in maternal blood [13], there have been concerted attempts to perform non-invasive prenatal testing (NIPT) with cffDNA [14]. The cffDNA is originated from apoptotic trophoblasts in the placenta [13]. On average, the amount of cffDNA in plasma from a pregnant woman is approximately 10% during 10–22 weeks of gestation but there is a large variance in the fraction of cffDNA between individuals [15]. The typical cffDNA size is approximately 150 base pairs (bp) [16]. The half-life of cffDNA is very short [17], and fetal DNA fragments of maternal blood are no longer detectable after birth [18]. Despite these limitations, NIPT consists of well-accepted and advanced technologies. NIPT has two key clinical advantages compared with those of invasive prenatal testing; it does not confer a risk of pregnancy loss, and can be performed during early pregnancy. However, NIPT is not currently considered to be fully diagnostic and requires invasive testing to confirm positive results because the discordant NIPT data for detecting chromosomal abnormality have resulted from placental or maternal cell mosaicism [19]–[21]. To minimize this problem, risk scores and multiple cut-offs have been often used for NIPT services [14].

Recent NIPT technologies are based on next-generation sequencing (NGS) [22]–[27]. NGS allows rapid and effective clinical diagnostic testing with two commonly used systems, the Illumina and Ion Torrent platforms. Ion Torrent, semiconductor sequencing platform, enables acquisition of sequencing data within 2 to 4 hours, thus the sequencer may provide an alternative clinical sequencing service with a reduced turn-around time. The majority of NIPT technology is associated with Illumina platform. Previous NIPT studies with the Illumina platform report consistent data for common aneuploidies, particularly trisomy 18 and 21; sensitivity and specificity are each higher than 98% [22]–[26]. A recent study reports the results for non-invasive detection of common fetal trisomy 13, 18, and 21 with Ion Proton, a semiconductor sequencer [27]. The sensitivity and specificity of the common fetal aneuploidies (Trisomy 13, 18, and 21) are each higher than 98% [27]. However, more references of non-invasive fetal aneuploidy detection with semiconductor sequencing platform are required.

In our study, we performed NIPT for fetal trisomy 18 and 21 with Ion Proton. The testing results of semiconductor sequencing platform have rarely been reported as an NIPT technology [27]. Therefore, we investigated whether fetal trisomy 18 and 21 were sensitively and specifically detectable by Ion Proton.

## Materials and Methods

### Study Subjects

From March 2012 to October 2013, we enrolled 155 pregnant women with fetuses who were diagnosed as high risk of fetal defects at Xiamen Maternal & Child Health Care Hospital (Xiamen, Fujian, China). 28 pregnant women (18.1%) were 12–16 weeks of gestation, 86 women (55.5%) were 17–21 weeks of gestation, and 41 women with fetuses (26.5%) were more than 22 weeks of gestation. Pregnant women who were scheduled to receive invasive diagnostic testing (Amniocentesis) for fetal karyotyping were asked to donate a blood sample before having the invasive test. Women who gave written informed consent participated in the study if they were ≥ 19 years old and had a singleton pregnancy with a gestational age of at least 12 weeks. The institutional review board at Xiamen Maternal & Child Health Care Hospital approved this study.

We used the results of standard prenatal aneuploidy screening with individual risk scores and interpretations produced by accredited clinical laboratories for sorting out high risk group of fetal defects. First-trimester serum markers included pregnancy-associated plasma protein A (PAPP-A) and free beta subunit or total human chorionic gonadotropin (hCG). Second-trimester serum markers included maternal serum alpha-fetoprotein (MSAFP), hCG, unconjugated estriol, and inhibin A. First-trimester serum markers were used in combination with sonographic measurement of fetal nuchal translucency (combined FTS test) to categorize patients into high or low risk groups. Second-trimester serum values were evaluated alone (quadruple screening test) or in combination with FTS results to define aneuploidy risk. To determine fetal karyotypes, fetal cells from amniotic fluid were cultured to test for chromosomal abnormalities by the method of Barch et al [28]. Cytogenetic analysis performed on all 155 fetal cells indicated that 139 (89.7%) were chromosomally normal, 5 (3.2%) were trisomy 18, and 11 (7.1%) were trisomy 21.

### Cell-free DNA Preparation and Sequencing

Eligible subjects were asked to participate in the study after receiving counseling about the invasive diagnostic test. Approximately 10 mL of whole blood was collected from each subject and placed in a Cell-Free DNA BCT™ tube (Streck, Omaha, NE, USA) before the invasive procedure. Within 6 hours of collection, the maternal blood samples were centrifuged first at 1,200×g for 15 min at 4°C. Plasma was transferred to microcentrifuge tubes, centrifuged again at 16,000×g for 10 min at 4°C to remove residual cells, transferred to fresh tubes, and stored at −80°C. For each sample, plasma cfDNA was extracted from 1 mL of plasma using the QIAamp Circulating Nucleic Acid Kit from Qiagen (Hilden, Germany). The resulting plasma cfDNA was used as input DNA to make a library for sequencing. End-repair of the plasma cfDNA was performed with T4 DNA polymerase, Klenow DNA polymerase, and T4 polynucleotide kinase. The adapter-ligated DNA libraries were analyzed with the Ion Proton™ System (Life Technologies, Grand Island, NY, USA) with an average 0.3×sequencing coverage per nucleotide. Ion PI™ Chip Kit version 2.0 (Life Technologies) was used for cfDNA sequencing and 10 cfDNA samples per chip were analyzed. Average total raw reads per sample was 6.5 million and mean rate of uniquely mapped reads was 59.0%.

### Data Analysis

Raw reads with different lengths acquired from the Ion Torrent Suite software trimmed from the 3′ end by sequencing quality value of >15, and filtered by read length (<50 bp) and GC contents (35%–45%). Duplicate reads were identified by Picard (http://picard.sourceforge.net/) with the default parameters and removed by in-house python script. The sequences were binned for each sample according to the index and mapped to the unmasked human genome sequence (hg19). We tested several mapping software programs, including BWA [29], Bowtie [30], and SOAP2 [31]. The results in this study were derived from the BWA mapping analysis. All chromosomes were divided into segments with a bin size of 300 kb to calculate z-score for fetal trisomy 18 and 21 detection. For the 155 samples, we calculated the z-score for each chromosome of each sample to examine fetal aneuploidy with mapped reads for each sample, and mean mapped reads and standard deviation (SD) of 139 euploid samples. For example, the z-score of case 1 for chr21 could be calculated as follows: z-score_chr21_case1_ = (mapped reads of chr21_case1_ – mean mapped reads of chr21_euploid group_)/(SD for mean mapped reads of chr21_euploid group_). The minimal z-scores to classify negative versus positive cases of trisomy based on interactive dot diagrams were >2.459 for fetal trisomy 18 and >2.566 for fetal trisomy 21. Sequence data has been deposited in the NIH short read archive (SRA) with the following BioProject accession number: SRP044689.

### Statistical Analysis

Categorical variables were summarized with the number and percentage of subjects. Continuous variables were described as means with SD. Positive predictive value (PPV) and negative predictive value (NPV) were calculated with standard formulas for binomial distributions. Wilson’s interval method was used to calculate 95% confidence intervals (CI). Interactive dot diagrams were used to assign classifications according to the presence of fetal aneuploidy. Analyses were performed with MedCalc version 12.1.4 (MedCalc Software bvba, Ostend, Belgium).

## Results

Table 1 shows the demographic characteristics of the enrolled participants in this study. The mean maternal age of this study cohort was 30.73±4.99 years (range 19–43 years). 28 pregnant women (18.1%) received NIPT during 12–16 weeks of gestation, 86 women (55.5%) received testing during 17–21 weeks of gestation, and 41 women with fetuses (26.5%) who were more than 22 weeks of gestation received testing. The ratio of male (50.3%) to female (49.7%) fetuses was nearly 1∶1; however, in aneuploidies, the ratio of male (81.3%) to female fetuses (13.8%) was 13∶3.

**Table 1:** Demographic characteristics of study subjects.

Demographic characteristics	Euploid (n = 139)	T18 (n = 5)	T21 (n = 11)	Total (n = 155)
Maternal age, years, mean±SD	30.61±5.01	27.60±5.50	33.64±3.29	30.73±4.99
≥ 35 years (%)	36 (25.9)	1 (20.0)	5 (45.5)	42 (27.1)
NIPT during 12–16 gestational weeks (%)	25 (18.0)	1 (20.0)	2 (18.2)	28 (18.1)
NIPT during 17–21 gestational weeks (%)	78 (56.1)	4 (80.0)	4 (36.4)	86 (55.5)
NIPT ≥ 22 gestational weeks (%)	36 (25.9)	0 (0.0)	5 (45.5)	41 (26.5)
Male fetus (%)	65 (46.8)	4 (80.0)	9 (81.8)	78 (50.3)
Female fetus (%)	74 (53.2)	1 (20.0)	2 (18.2)	77 (49.7)
Z-score of chr18 (min, max)	−3.184, 2.459	4.017, 10.193	−1.730, 1.592	−3.184, 10.193
Z-score of chr21 (min, max)	−3.080, 2.566	−1.449, 0.387	4.693, 30.943	−3.080, 30.943

T18, Trisomy 18; T21, Trisomy 21; SD, standard deviation; NIPT, non-invasive prenatal testing.

**Figure 1:**
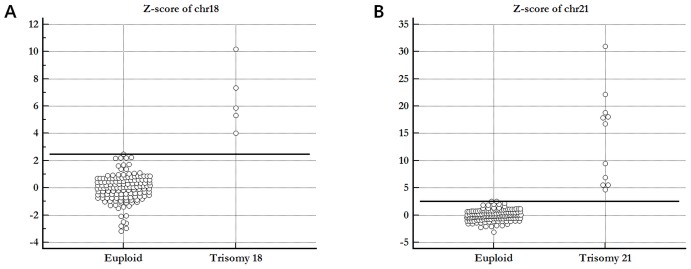
Interactive dot diagrams of trisomy 18 and 21. (A) Interactive dot diagram of trisomy 18. (B) Interactive dot diagram of trisomy 21.

Figure 1 presents interactive dot diagrams for fetal trisomy 18 and 21 showed the minimal z-score values of 100% PPV and NPV. For fetal trisomy 18, the minimal z-score value of 2.459 showed 100% PPV and NPV. The minimal z-score of 2.566 was used to classify negative versus positive cases of fetal trisomy 21.

Table 2 summarizes the PPVs and NPVs of the NIPT results for fetal trisomy 18 and 21. For fetal trisomy 18, the PPV (95% CI: 47.8%–100.0%) and NPV (95% CI: 97.6%–100.0%) were 100%. The PPV (95% CI: 71.5%–100.0%) and NPV (95% CI: 97.5%–100.0%) for detecting fetal trisomy 21 were also 100%. On the whole, the PPV (95% CI: 79.4%–100.0%) and NPV (95% CI: 97.4%–100.0%) of combined detection of fetal trisomy 18 and 21 were 100%.

**Table 2:** Positive predictive and negative predictive values in this study.

Aneuploidy	Positive predictive value (95% CI)	Negative predictive value (95% CI)
T18 (n = 5)	100.0% (47.8%–100.0%)	100.0% (97.6%–100.0%)
T21 (n = 11)	100.0% (71.5%–100.0%)	100.0% (97.5%–100.0%)
Combined detection (n = 16)	100.0% (79.4%–100.0%)	100.0% (97.4%–100.0%)

T18, trisomy 18; T21, trisomy 21.

## Discussion

We have examined the feasibility of NIPT using cffDNA in a predominantly high-risk obstetrics population using the semiconductor sequencing system: Ion Proton. Fetal trisomy 18 and 21 were non-invasively detectable with 100% PPV and NPV. The results of present study provide the evidence that non-invasive fetal trisomy 18 and 21 detection can be performed with semiconductor sequencing platform. However, our test is still far away from a clinically validated test because there was not consideration for plasma fetal DNA fraction.

According to previous reports, the Ion Torrent sequencer shows equivalent performance to the Illumina platform [32]–[34]. Quail et al. [32] measured Ion Torrent PGM data against Illumina HiSeq2000 and MiSeq results, and found that the error rate of Ion Torrent PGM data was 1.78%. In contrast, Illumina HiSeq2000 and MiSeq platforms demonstrated error rates ranging from 0.26% to 0.80%, and the rates of true single nucleotide polymorphisms (SNPs) were higher for Ion Torrent PGM analysis compared to that of the Illumina HiSeq2000 and MiSeq analysis [32]. Boland et al. [33] demonstrated that Ion Proton genotyping data were nearly concordant with SNP microarray data; further, the concordance rates were similar to those of the Illumina HiSeq2000 results. Chen et al. [34] compared the performances of the Ion Proton and Illumina MiSeq systems for ultra-low coverage sequencing. Ion Proton and Illumina MiSeq platforms present similar coverage for relative depth (RD) at each 1-Mbp of sequencing for GC-content. Both sequencing systems correctly detected fetal tissue aneuploidies [34]. Ion Proton system can construct ∼80 million raw reads in 3–4 hours, which allows chromosomal abnormality detection within 1–2 working days, is advantageous for applications that require precise turn-around times, such as prenatal genetic screening [34].

Conventional noninvasive aneuploidy screening tests are not straight forward. None of the screening tests are ideal, because detection rates for chromosomal abnormalities are less than 100%. By definition, the false-positive rate is 5% if modeled for a common obstetrics population. Maternal serum marker and ultrasound screening tests have been performed widely for over a decade. These have been popular but have limitations. Henry et al. [35] analyzed the birth of Down’s syndrome after routine noninvasive screening largely replaced age-related invasive procedures in Colorado from 1989 to 2005. Despite the increase in prenatal screening, neonates with trisomy 21 increased in women over 35 years of age. One explanation is that women did not receive the recommended protocol, perhaps incorrectly assuming that a single blood test would definitively detect abnormalities.

Serum biomarker analysis and ultrasound sonography do not definitively detect chromosomal aneuploidy. A positive screen result must be followed up with an invasive prenatal test (CVS or amniocentesis) to determine whether fetal aneuploidy is truly present. Previous studies of NIPT with cffDNA report consistently accurate results for detecting common aneuploidies, particular trisomy 18 and 21 [22]–[27]. However, the cost of the test is a major issue. Currently, the cost of a plasma cffDNA test ranges from about $800 to $2,000 in the US and from $500 to $1,500 in other countries [14]. Two previously published reports have estimated costs when the cfDNA test is applied to women with positive conventional screening results [36], [37]. They concluded that applying the cfDNA test was connected to a net cost reduction in comparison with CVS or amniocentesis [36], [37].

In summary, we investigated whether fetal trisomy 18 and 21 could be detected by noninvasive Ion Torrent sequencing as has previously been published for Illumina sequencing. However, the present study has several limitations. First, our sample size was smaller than other obstetrics populations in previous reports [22]–[27]. The lower 95% CIs for NPV were almost 97.0% in our study, whereas other studies usually demonstrate >98.0% of the lower 95% CIs for specificity. More than 400 high-risk pregnant women will need to be enrolled in future studies to estimate >98.0% of the lower 95% CIs for NPV or specificity with Wilson’s interval method. Second, we did not find any positive cases for rare chromosomal abnormalities, such as trisomy 9, trisomy 13, and sex chromosome aneuploidies. Third, the participation rates for low-risk pregnant women after serum biomarker testing and ultrasound sonography did not reach expected levels. Fourth, we could not measure Ion Proton sequencing data against data that were analyzed by Illumina HiSeq or MiSeq due to insufficient quantities of blood samples. Although the results of our study provide initial evidence for NIPT of fetal trisomy 18 and 21 using the semiconductor sequencing system, a prospective study on a larger cohort of clinically diverse obstetrics participants is warranted to validate these findings.
